# Βeta-fibrinogen gene promoter A −455 allele associated with poor longterm survival among 55–71 years old Caucasian women in Finnish stroke cohort

**DOI:** 10.1186/1471-2377-14-137

**Published:** 2014-06-22

**Authors:** Mika Martiskainen, Niku Oksala, Tarja Pohjasvaara, Markku Kaste, Anni Oksala, Pekka J Karhunen, Timo Erkinjuntti

**Affiliations:** 1 School of Medicine, University of Tampere and Fimlab Laboratories, Pirkanmaa Hospital District, 33014 Tampere, Finland; 2 Division of Vascular Surgery, Department of Surgery, Tampere University Hospital, Tampere, Finland; 3 Department of Neurology, Helsinki University Central Hospital, Helsinki, Finland

**Keywords:** Adult, Cohort study, Cerebral infarction, Stroke, Risk factors, Genetic and inherited disorders, Genetics, Genetic polymorphisms, Fibrinogen, Haematological disorders

## Abstract

**Background:**

Women die of stroke more often than men. After menopause, the incidence of ischemic stroke increases rapidly. Elevated fibrinogen levels and smoking have been associated with an increased risk of stroke. In gene-cluster haplotype analyses, the beta-fibrinogen (FGB) promoter −455 G/A polymorphic locus was most strongly associated with elevated plasma fibrinogen levels. We investigated whether the FGB −455 G/A polymorphism and smoking might interact with sex on longterm survival of acute stroke sufferers.

**Methods:**

The Stroke Aging Memory (SAM) cohort comprising 486 consecutive stroke patients (55–85 years, 246 men, 240 women) subjected to clinical and MRI examination was followed over 12.5 years. During this period 347 (71.4%) patients died. The genotypes of the FGB −455 G/A polymorphism were determined by PCR.

**Results:**

The FGB −455 G/A polymorphism genotype distributions were 64.7%, 32.1%, and 3.2% for GG, GA, and AA, respectively. During the follow-up, the FGB −455 A + genotype did not associate with survival, nor was there any genotype-by-smoking interaction on poor outcome in the total study population. However, women aged 55–71 years who carried the FGB −455 A-allele showed worse survival regardless of smoking status compared to non-smoking FGB −455 GG homozygotes (non-smokers, crude HR = 5.21, 95% CI: 1.38-19.7; smokers, crude HR = 7.03, 95% CI: 1.81-27.3). This association persisted in adjusted analyses. No such association was observed for women in the oldest age-group, nor among men.

**Conclusion:**

The A + genotype of the FGB −455 G/A polymorphism associated with poor survival among 55–71 years old Caucasian women in the Finnish stroke cohort.

## Background

Cardiovascular and cerebrovascular diseases are major causes of death in industrialized countries, and stroke is the leading cause of adult disability in the developed world [1,2]. While combined death rates and hospitalization periods of cardiovascular diseases due to myocardial infarction have decreased greatly, the numbers related to stroke have decreased only slightly. In addition to family history and lifestyle factors, gene-environment interactions seem to determine the development and manifestation of cardio- and cerebrovascular diseases through inflammatory and prothrombotic processes [3-7].

Despite a lower incidence of stroke over one’s lifetime, women are twice as likely to die from stroke as men and during menopause the incidence of ischemic stroke increases rapidly [8-10]. Postmenopausal women suffer more severe and fatal strokes indicating the presence of possible unknown sex-specific and genetic factors [11]. Possible approaches to the search for genetic factors of stroke are genome-wide association studies (GWAS) and candidate gene studies. While GWAS have uncovered numerous associations with metabolic endpoints, it has been more challenging to discover associations with complex diseases in spite of the formation of large consortia [12]. An alternative to GWAS is the investigation of candidate genes based on knowledge of risk factors or the pathophysiology of the disease.

Fibrinogen is a dimeric 340 kDa acute phase glycoprotein synthesized by the liver. It consists of three polypeptides Aα, Bβ and γ coded by the alpha (FGA), beta (FGB) and gamma (FGG) genes, respectively [13]. Fibrinogen is an important component of the coagulation cascade and a major determinant of blood viscosity and platelet aggregation. It modulates endothelial function and promotes smooth muscle cell proliferation and migration [4,7]. Smoking increases blood fibrinogen concentration and is a significant risk factor for stroke [14-16]. Recently, obesity has been associated with elevated fibrinogen and CRP levels [17].

In the SAM cohort, we have previously found a genotype-by-smoking interaction between the A-allele of the FGB −455 G/A polymorphism and recurrent lacunar infarcts, as well as an interaction between smoking and the blood platelet fibrinogen receptor (GpIIb/IIIa) PlA2 allele as a risk factor for lacunar cerebral infarction [18,19]. In haplotype analyses covering the entire FGB-gene, the FGB −455 G/A polymorphism (rs1800790) shows the strongest association with blood fibrinogen levels, although a direct relationship with atherothrombotic disease is lacking [6,20-25].

The A + genotype of the FGB −455 G/A polymorphism acts as an individual risk factor for coronary artery disease (CAD), acute coronary syndromes (ACS), peripheral arterial disease (PAD) and stroke [7,14,26-30]. In the present study our hypothesis is that the FGB −455 G/A polymorphism might interact with smoking and interfere sex specific longterm survival after stroke.

## Methods

### Patients

The Helsinki Stroke Aging Memory (SAM) cohort comprises of a consecutive series of all Finnish (Caucasian) patients with suspected stroke admitted to Helsinki University Central Hospital (n = 1622) between the 1^st^ of December 1993 and the 30^th^ of March 1995. A total of 486 (246 men, 240 women) living patients aged 55 to 85 years (mean age 71.3 years) were recruited for the study 3 months after experiencing an ischemic stroke - more detailed descriptions of the study series and methods have been described elsewhere [17,18,31,32].

### General clinical assessment

A structured medical and neurological history was recorded during hospitalization for the index stroke that was also the time point of laboratory sample collection and blood pressure measurements in addition to MRI investigation. History of cardiac risk factors (myocardial infarction, cardiac failure, atrial fibrillation), arterial hypertension, peripheral arterial disease, and diabetes was investigated by reviewing all available hospital charts, in addition to a structured interview of the subject and a knowledgeable informant. Hypertension was defined as blood pressure ≥160/95 mm Hg. Smoking habit was scored as non-smokers or smokers, with non-smokers only those who had never smoked. Diabetes was defined as previously documented diagnosis, current use of insulin or oral antidiabetic medication, or fasting blood glucose >7.0 mmol/L. Laboratory analyses included total and HDL cholesterol, triglycerides, and fasting blood glucose. Total cholesterol was considered high at >6.5 mmol/L. Carotid stenosis was defined as >50% stenosis of vessel diameter. 383 patients (78.8%) of the SAM cohort underwent a brain MRI investigation, and 371 patients had both MRI and genotype data. Infarct subtype definitions for lacunar (LAI) and large-vessel infarcts (LVI) have been presented earlier [33]. There were 116 (23.8%) of patients with missing values in MRI, cause of death, biochemical or genotype data. The final follow-up study population did not differ in risk factor, stroke type, demographic, or medical parameters from the original cohort. The analyzed and excluded patients did not differ in either vascular risk factors or stroke type (Table 1). Data on survival were collected over a 12.5 year period during which 347 (71.4%) of the patients deceased. MRI, genotype and cause of death data was available for 262 (53.9%) patients. The study was approved by the ethics committee of the Helsinki University Central Hospital, Helsinki, Finland. The study was explained to the patients, and informed consent was obtained.

**Table 1:** Main characteristics of 371 patients in the SAM cohort with survival and genotype data according to -455G/A genotypes

**Variable**	**FGB −455 G/A genotype**	
	**G/G (n = 240)**	**G/A + A/A (n = 131)**
Age (mean, SD), y	71.1 (7.67)	71.0 (8.10)
Women, %	48.3	53.4
CRP >10 mg/L, %	18.3	16.7
Diabetes, %	23.8	21.4
Hypertension, %	47.1	50.4
Hypercholesterolemia, %	15.4	17.6
Mean fs-Chol (SD)	5.56 (1.14)	5.57 (1.23)
Mean fs-Chol-HDL (SD)	1.15 (0.34)	1.15 (0.34)
Hypertrigyceridemia, %	2.1	3.1
Smoking, %	48.5	53.8
Subtypes of ischemic stroke		
Large artery, %	17.3	18.1
Cardioembolic, %	5.4	8.4
Lacunar, %	15.4	11.5
Other, %	62.1	61.8
MI, %	17.9	19.1
Arrhythmia, %	24.2	24.4
Carotid artery stenosis, %	12.5	10.7
Atrial fibrillation, %	17.2	20.6

### DNA preparation

DNA was separated from frozen blood samples according to standard procedures. The genotypes of the FGB −455 G/A polymorphism were detected by polymerase chain reaction (PCR) and restriction enzyme digestion, followed by poly-acrylamide gel electrophoresis. Primer sequences and the PCR protocol have been previously described in detail [34].

### Statistical analysis

Data was analysed using SPSS/WIN (version 20.0, SPSS Inc.) software. The number of A/A homozygotes of the FGB −455 G/A polymorphism was small among SAM cohort patients and therefore A/A homozygotes and G/A heterozygotes were grouped together to form the A + genotype variable of the of the FGB −455 G/A polymorphism. The data was analysed as whole and in subgroups that were formed using sex and the mean age (71 years) to divide the data into age groups (55–71 and 72–85 years old) in intention to study the sex and age dependence. To study the association between risk factors including FGB −455 G/A A + genotype and survival we first analysed the data by Kaplan-Meier (log rank) survival analysis and graphical approach was used in verification that the proportional hazards assumption was met. Subsequently, the Cox regression proportional hazards model was used to study the association between FGB −455 G/A A + genotype and survival. First we obtained crude hazard ratios (HRs) and confidence intervals (CIs) and then we used multivariate Cox regression analysis in further statistical testing of genotype-survival association with age, history of previous arrhythmias, atrial fibrillation (AF), carotid stenosis (CS), elevated CRP (>10 mg/l), diabetes, hypercholesterolemia, hypertension, hypertriglyceridemia, history of myocardial infarction (MI), sex and smoking pooled into forced and forward stepwise likelihood ratio (LR) models. To further adjust the analyses we stratified Cox regression models (forced and forward stepwise) with each modifier (history of previous arrhythmias, AF, CS, elevated CRP (>10 mg/l), diabetes, hypercholesterolemia, hypertension, hypertriglyceridemia, history of MI and smoking) separately and used age and sex as confounders. The data concerning the history of arrhythmias, AF, CS, diabetes, hypertension, hypercholesterolemia and hypertriglyceridemia was missing in 118 (24.3%) of patients. Furthermore, based on Little’s MCAR (missing completely at random) test (p < 0.05) we concluded that the data was not missing completely at random. Overall summary of missing values showed that each case with missing values had, on average, missing values on roughly 3.37 of the 10 variables suggesting that listwise deletion would lose much of the information in the data. The data missingness pattern was nonmonotone and the random number generator was used to simulate missing values for each observation that were imputed with fully conditional specification (FCS) Markov Chain – Monte Carlo (MCMC) method assuming multivariate normality and five imputations was suitable in graphic check for FCS convergence. Then we analyzed the complete data with Cox regression model to check differences in survival in total population and in subgroups with pooled parameter estimates and we also checked of the variation in the regression coefficient estimates from imputation to imputation, and against the original data. The results were not altered after imputations. Survival analyses for LAI or LVI subgroups, stroke types (TOAST) and specific causes of death were also completed. According to power calculations our study population has the power to show hazard ratios >2.5 to be statistically significant with 0.80 probability (power) when the Type I error probability is 0.05 [35].

## Results

### Prevalence of FGB −455 G/A alleles

There were 371 patients with the FGB −455 G/A polymorphism genotype and clinical data available. The study population comprised 50.7% (186) male patients. The mean age was 71.0 years (SD ± 7.81) and the data was normally distributed. There were 186 smokers (50.1%) in the study population (84.9% men and 15.1% women). Allele distributions were in Hardy-Weinberg equilibrium (p = 0.74). Genotype distributions were 64.7% for GG, 32.1% for GA, and 3.2% for AA. These frequencies closely correspond to the population frequencies among whites; in the ECTIM study, the allele frequencies were 65.9%, 29.1%, and 4.9%, respectively [27]. The main demographic parameters are represented in Table 1.

### Causes of death

Of the series 262 (70.6%) patients with genotype and MRI data died during an average of 12.5 years of follow-up. Cardiovascular diseases were the cause of death in the majority of cases (33.6% of men and 30.5% of women). There were no statistically significant differences between sexes in the causes of death; 37.7% of women and 29.5% of men had brain related causes of death and 34.6% of women and 27.3% of men had ischemic brain insults. However, 72–85 years old men had more often brain related (35.4% vs. 20.8%, p = 0.007) and ischemic brain insults (32.9% vs. 18.9%, p = 0.01) as cause of death compared to 55–71 years old men (Table 2). In addition, ischemic brain insults and cardiovascular diseases were more often the cause of death among women aged 72–85 years compared to younger age group (36.7% vs. 23.8%, p = 0.06; 33.9% vs. 19.0, p = 0.04, respectively). Furthermore, cerebral bleeding was the cause of death more often among 55–71 years old women compared to older age group (14.3% vs. 2.8%, p < 0.001) (Table 2).

**Table 2:** Causes of death according to age group (55–71 and 72–85 years) and sex during longterm post stroke follow-up in the SAM cohort

**Cause of death**	**Valid data**	**All**		**Men**	**Men**	**Women**	**Women**
	**n**	**55-71 yrs**	**72-85 yrs**	**55-71 yrs**	**72-85 yrs**	**55-71 yrs**	**72-85 yrs**
		**n = 74**	**n = 188**	**n = 53**	**n = 79**	**n = 21**	**n = 109**
		**(28.2%)**	**(71.8%)**	**(40.2%)**	**(59.8%)**	**(16.2%)**	**(83.8%)**
**Brain related**	262	18 (24.3)	70 (37.2)	11 (20.8)	28 (35.4)	7 (33.3)	42 (38.5)
**Ischemic**	262	15 (20.3)	66 (35.1)	10 (18.9)	26 (32.9)	5 (23.8)	40 (36.7)
**Bleeding**	262	4 (5.4)	6 (3.2)	1 (1.9)	3 (3.8)	3 (14.3)	3 (2.8)
**Dementia**	262	3 (4.1)	5 (2.7)	1 (1.9)	4 (5.1)	2 (9.5%)	1 (0.9)
**Cardiac**	262	25 (33.8)	55 (29.3)	21 (39.6)	18 (22.8)	4 (19.0)	37 (33.9)
**Cancer**	262	15 (20.3)	23 (12.2)	9 (17.0)	14 (17.7)	6 (28.6)	9 (8.3)
**Infection**	262	1 (1.4)	10 (5.3)	1 (1.9)	5 (6.3)	0 (0.0)	5 (4.6)
**Trauma**	262	4 (5.4)	6 (3.2)	4 (7.5)	3 (3.8)	0 (0.0)	3 (2.3)
**Other**	262	10 (13.5)	22 (11.7)	7 (5.3)	10 (12.7)	3 (14.3)	12 (11.0)

All figures in parentheses are percentages.

From the categories ‘all’ and men, 1 case was excluded as cause of death could not be specified.

### Long-term survival

As expected, age was associated (p < 0.001) with survival in Kaplan-Meier log rank analyses. In the multivariate Cox regression model, age (HR = 1.08, 95% CI: 1.06-2.00, p = 0.005) and diabetes (HR = 1.39, 95% CI: 1.05-1.83, p = 0.03) associated significantly with poor survival. However, significant association between diabetes and risk of cardiac death (HR = 1.59, 95% CI: 0.97-2.59) was not observed in multivariate Cox regression analysis. Smoking had no statistically significant (p = 0.06) effect on survival in the Kaplan-Meier log rank analysis that was also observed in the multivariate Cox Regression model (HR = 1.26, 95% CI: 0.97-1.65) and it was not dependent on sex. In contrast, hypercholesterolemia or elevated cholesterol levels known to increase risk for further cardiovascular events, but paradoxically associates with lower mortality in old age had a positive (HR = 0.65, 95% CI: 0.43-0.99, p = 0.05) impact on longterm post stroke survival in Cox regression model [36].

**Figure 1:**
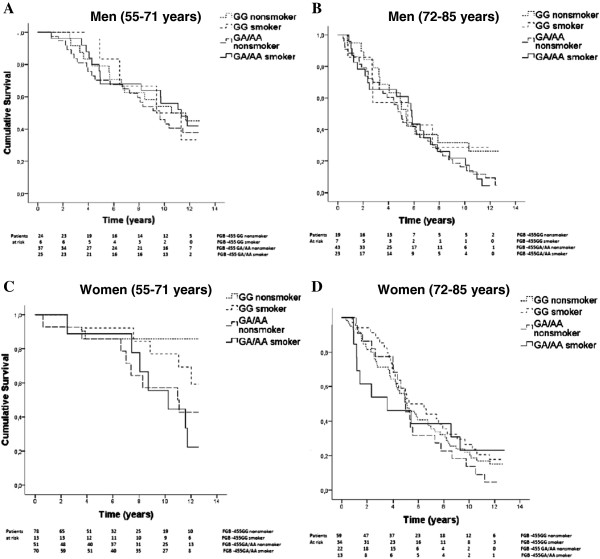
**The effect of FGB -455G/A genotype-by-smoking interaction on overall post stroke survival (all cause death endpoint) in the SAM cohort.** Data shown on men (**A**: 55–71, and **B**: 72–85 years old); and women (**C**: 55–71, and **D**: 72–85 years old). Survival curves were obtained by Kaplan-Meyer log rank analysis that showed poorer longterm survival in the 55–71 year old women who carried the FBG -455G/A A-allele. This association was not dependent on smoking status in Cox regression forced model (non-smokers crude HR: 5.21, 95% CI 1.38-19.7; smokers crude HR: 7.03, 95% CI 1.81-27.3). There were no significant associations with Kaplan-Meyer (log rank) or Cox regression analysis between interaction terms and survival among men in both age groups or among older women (72–85 years).

**Figure 2:**
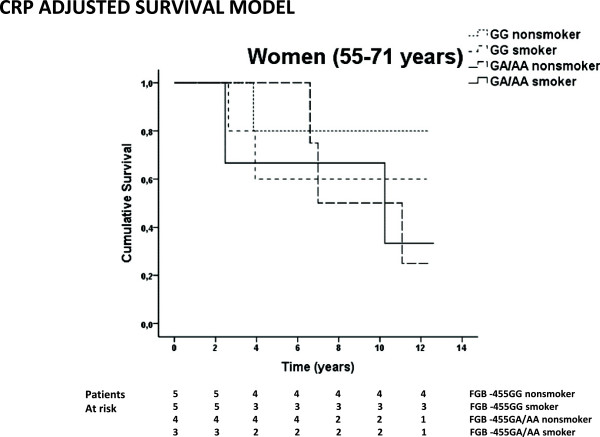
**The effect of FGB -455G/A genotype-by-smoking interaction adjusted for elevated CRP on overall post stroke survival (all cause death endpoint) in the 55–71 year old women of the SAM cohort.** Survival curves were obtained by Kaplan-Meyer log rank analysis showed poorer longterm survival in the 55–71 year old women who smoked (p = 0.002). Hazard ratios and 95% confidence intervals were obtained by Cox regression forced model adjusted for elevated CRP and the progressive effect of smoking and FGB −455 G/A polymorphism on worse survival was observed ( GG smokers, adjusted HR: 9.83, 95% CI 1.20-80.1; GA/AA non-smokers, adjusted HR: 15.4, 95% CI 1.91-124; GA/AA smokers, adjusted HR: 24.9, 95% CI 3.00-206).

We did not observe statistically significant (p-values >0.05) association between the FGB −455 G/A A-allele and poor survival in the total study population, nor in men or women separately (Kaplan-Meier log rank and Cox regression model). In addition, genotype-by-smoking interaction on survival in the total study population was not observed in the Kaplan-Meier log rank analysis that remained insignificant in Cox Regression model (Table 3). However, patients with FGB −455 G/A A + genotype of the younger age group (55–71 years old) had worse survival (non-smoker HR = 2.37, 95% CI: 1.24-4.41; smoker HR = 2.33, 95% CI: 1.20-4.52) despite of smoking status suggesting an association between the carrier status of the FGB −455 G/A A-allele and elevated risk for death (Table 3). In subgroup analysis we detected a significant association between the A + genotype of the FGB −455 G/A polymorphism and worse post stroke survival in 55–71 year old women in Kaplan-Meier (p = 0.013) log rank analysis and multivariate Cox Regression forced model (non-smoker, crude HR = 5.21, 95% CI: 1.38-19.7, p = 0.015; smoker, crude HR = 7.03, 95% CI: 1.81-27.3, p = 0.005) (Figure 1 and Table 3). When history of arrhythmias, AF, CS, CRP, diabetes, hypercholesterolemia, hypertension, hypertriglyceridemia, and history of MI were forced into Cox regression model, the association between FGB −455 A-allele and worse survival persisted, however wider CIs were observed reflecting smaller sample size (non-smoker HR = 41.0, 95% CI: 3.23-520, p = 0.004; smoker HR = 37.2, 95% CI: 2.50-548, p = 0.009). Similar results was obtained in Cox regression forward stepwise LR (likelihood ratio) model (non-smoker HR = 26.9, 95% CI: 2.58-279, p = 0.006; smoker HR = 36.1, 95% CI: 3.42-381, p = 0.003) (Figure 1). In Cox regression analysis with stratification for each modifier showed that elevated CRP strengthened the effect of smoking and FGB −455 G/A polymorphism on worse survival in women aged 55–71 years (GG smokers, adjusted HR: 9.83, 95% CI 1.20-80.1; GA/AA non-smokers, adjusted HR: 15.4, 95% CI 1.91-124; GA/AA smokers, adjusted HR: 24.9, 95% CI 3.00-206), however the number of patients decreased considerably in this model (Figure 2). This association was not seen in 72–85 years old women or in men. In addition, we found no genotype-by-smoking interaction with specified cause of death in any age group of men or women.

**Table 3:** Cox regression analysis of the association of the FGB −455 G/A genotype-by-smoking interaction term with longterm survival (all cause death endpoint) among patients with ischemic stroke of the SAM cohort (HRs and CIs are represented as crude)

**FGB −455 G/A carrier and smoking status**	**All**	**Age group**	**Age group**	**Men**	**Women**	**Men**	**Women**
	**HR 95% CI**	**55–71 years**	**72–85 years**	**(55–71 years)**	**(55–71 years)**	**(72–85 years)**	**(72–85 years)**
		**HR 95% CI**	**HR 95% CI**	**HR 95% CI**	**HR 95% CI**	**HR 95% CI**	**HR 95% CI**
**All n**	369	165	204	104	61	80	124
**Event n**	262	90	172	64	25	69	104
**GG nonsmoker**	Ref.	Ref.	Ref.	Ref.	Ref.	Ref.	Ref.
**GG smoker**	1.03 (0.71-1.49)	1.35 (0.65-2.80)	0.91 (0.59-1.41)	1.07 (0.39-2.94)	3.46 (0.89-13.4)	1.38 (0.45-4.31)	0.83 (0.52-1.34)
**GA/AA nonsmoker**	1.34 (0.99-1.81)	2.00 (1.13-3.54)	1.27 (0.88-1.81)	1.21 (0.64-2.30)	5.21 (1.38-19.7)	1.62 (0.84-3.12)	1.17 (0.70-1.95)
**GA/AA smoker**	1.18 (0.83-1.67)	2.03 (1.11-3.70)	1.11 (0.70-1.75)	1.10 (0.56-2.17)	7.03 (1.81-27.3)	1.57 (0.74-3.30)	0.93 (0.47-1.84)

Data shown for the total population and for, subgroups based on age (55–71 and 72–85 years old) and further divided by sex.

## Discussion

The A + genotype of the FGB −455 G/A polymorphism was associated with poor post stroke survival among 55–71 years old postmenopausal women of the Stroke Aging Memory (SAM) study cohort, representing a consecutive series of all Finnish (Caucasian) patients with suspected stroke admitted to Helsinki University Central Hospital during 16 months in 1993–1995. This association was not observed in older women of the cohort or among men.

In our study, the significant association between FGB −455 G/A polymorphism and survival in total study population was not observed. Pruissen et al. previously studied the impact of FGB −455 G/A polymorphism and other prothrombotic polymorphisms on composite outcome events after cerebral ischemia of arterial origin [37]. They found that FGB −455 G/A polymorphism did not associate with survival that is in line with our results when the whole series was studied without sex or age dependences. The lack of significant association between the A + genotype of the FGB −455 G/A polymorphism and poor survival among older patients (72–85 years) may be explained by the burden of other illnesses and old age predisposing to poor outcome. However, our finding on the association of FGB −455 G/A A-allele with poor survival is restricted only to postmenopausal women of age 55 to 71 years for we had no premenopausal women included into the SAM cohort. In addition, we found that 34.6% of women and 27.3% of men had ischemic brain insults as the cause of death that supports the concept that women are at greater risk of stroke after menopause (Table 2). However, we did not observe any genotype-by-smoking interaction with causes of death, although smoking is known to increase fibrinogen levels.

During menopause, the protective effect of estrogen against stroke and cardiovascular diseases reduces and hormone replacement therapy (HRT) used to treat the symptoms of menopause acts as a risk factor for stroke [38-40]. Based on the data of The Finnish National Health Institute, 32.0% of women aged 55–70 years and 11.0% of women aged 71–85 years used HRT during the year 1996. Furthermore, 40.9% of women aged 55–64 years used HRT in Finland during the year 1999 [41]. The exact statistics concerning HRT use in the Helsinki region during the early 1990’s could not be obtained as national computer-based databases of pharmaceutical store sales were gradually taken into use in Finland only in the late 1990’s. As HRT is exclusively used in the group of 55–70 year old women, in contrast to those at the age of 71–85 we may speculate that our results might be partly explained by a synergy between the FGB −455 A + genotype, smoking and HRT. On the contrary, transdermal estradiol therapy may have antiatherosclerotic effects in postmenopausal women by improving vascular atherosclerosis [42]. Matsui S et al. have found that HRT regimen using oral ultra-low-dose estradiol and dydrogesterone has an improving effect on arterial stiffness and insulin resistance in 28 postmenopausal women [43]. As we do not have data on medication history that is an important limitation of our study, the potential effect of HRT on stroke risk or survival could not be evaluated. Furthermore, HRT may act as a strong confounding factor and have an impact on the results.

The SAM cohort with study subpopulations are limited in size and the main finding of positive association of FGB −455 G/A A-allele with poor survival is based on a subgroup of only 21 women aged 55–71 years old that complicates the interpretation of the results. However, this association without the smoking status dependence was already observed in 55–71 years old patients of the SAM cohort (Table 3). In addition, the SAM cohort is too small to allow the detailed analysis of all fibrinogen haplotypes and their impact on survival. One limitation in our study is that the blood or plasma fibrinogen levels were not determined. However the A-allele of the FGB −455 G/A polymorphism has been shown to associate with elevated fibrinogen levels and possess functional properties [44].

Overweight/obese women without clustering of cardiometabolic risk factors possess abnormal levels of inflammatory markers including CRP and fibrinogen suggesting that obesity represents a chronic inflammatory state [17]. We could not assess the effect of obesity on survival. In our cohort adjustment for elevated CRP (>10 mg/L) value as a marker of inflammation caused a significant increase in the point HR estimates addressing the significant association of FGB −455 G/A A + genotype towards poor survival. However, the CIs widened substantially that reflects the small number of patients in this subgroup analysis (Figure 2). Therefore the interaction between FGB −455 G/A polymorphism and elevated CRP remains unclear. In recent study of Ock SY et al. the elevated plasma CRP concentration were discussed to be a reliable surrogate marker for predicting carotid atherosclerosis severity in patients with AF, and that CRP concentration may be related to an increased risk of ischemic stroke [45]. In addition, CRP activity has been positively correlated with plaque instability as well as intima to media wall thickness of coronary arteries and the common carotid artery [46,47]. Concerning these, ischemic stroke is a complex trait with polygenic and multifactorial inheritance with environmental triggers [48,49]. Gene-environment-life style interactions influence the risk for stroke in an individual, and based on our findings these may modify the survival from stroke.

Misclassification of the exposure is unlikely since medical history and risk factor data were derived from administrative hospital data and genotyping was made in the accredited laboratory. The stroke diagnosis and classification were based on MRI and patients were assessed by a senior neurologist that diminishes misclassification of the outcome. In addition, the possibility of a selection bias may underestimate the proportion of women who died before hospital assessment at 3 months. Furthermore, the initiation of antithrombotic, antihypertensive and lipid treatments poststroke may attenuate the effect of genetic factors on survival. Limitations of the cohort are also discussed in previous publication by Oksala N et al. [19].

## Conclusions

Taking in account the limitations of the present study, our study highlights the contribution of the A + genotype of the FGB −455 G/A polymorphism towards poor poststroke survival among 55–71 years old Caucasian women even after adjustment for arrhythmias, AF, CS, CRP, hypercholesterolemia, hypertension, hypertriglyceridemia, and history of MI with age and sex as confounding factors. This association was not observed in older women or among men. The SAM cohort does not comprise stroke patients under 55 years old and the association of FGB −455 G/A polymorphism with poor survival could not be assessed in premenopausal women. Furthermore, we found a substantial interaction in the adjusted risk of death attributable to FGB −455 G/A A + genotype and elevated CRP that warrants the further investigation of possible gene-environment-life-style interactions.

## Competing interest

The authors declare that they have no competing interests.

## Authors’ contributions

MM participated in the conception and design of the study, acquisition of data, analysis and interpretation of data, drafting of the manuscript, critical revision of the manuscript, administrative, technical, and material support. NO participated in the conception and design of the study, analysis and interpretation of data, critical revision of the manuscript and supervision. TP participated in the conception and design of the study, acquisition of data and critical revision of the manuscript. MK participated in the conception and design of the study, acquisition of data, critical revision of the manuscript, administrative, technical, and material support, supervision, and obtaining funding. AO participated in acquisition of data, and critical revision of the manuscript. PK participated in acquisition of data, supervision and critical revision of the manuscript. TE participated in the conception and design of the study, acquisition of data, critical revision of the manuscript, administrative, technical, and material support, supervision, and obtaining funding. All authors read and approved the final manuscript.

## Pre-publication history

The pre-publication history for this paper can be accessed here:

http://www.biomedcentral.com/1471-2377/14/137/prepub
